# Whether specific genetic feature predicted immunotherapy efficacy: A case report

**DOI:** 10.1097/MD.0000000000036922

**Published:** 2024-01-12

**Authors:** Jun Chen, Linrong Pang, Lianxiang He, Ting Li, Xiaochun Cheng

**Affiliations:** a Cancer Chemoradiotherapy Center, The Affiliated People’s Hospital of Ningbo University, Ningbo, China; b Medical Affairs Department, Guangzhou Gloria Biosciences Co. Ltd., Beijing, China.

**Keywords:** case report, cervical adenocarcinoma, immunotherapy, potential biomarker

## Abstract

**Rationale:**

Blockade of programmed death protein 1 (PD-1), have been observed to have quite good efficacy in recurrent and metastatic cervical cancer. Generally, we believe that the biomarkers of PD-1 inhibitors are programmed cell death-ligand 1, tumor mutational burden, high microsatellite instability, or deficient mismatch repair. However, in the case reported below, we observed that the patient with negative existing predictive biomarkers have significant benefits after zimberelimab monotherapy, indicating that there were other biomarkers that may predict immunotherapy efficacy. However, currently, no one has explored and studied the other potential biomarkers of PD-1 inhibitors.

**Patient concerns:**

A 51-year-old patient, diagnosed with cervical adenocarcinoma nearly 11 years ago, requested treatment.

**Diagnoses:**

The next-generation sequencing has shown PIK3CA E545K, SMAD4 1309-1G, and ALK E717K gene mutations, receptor tyrosine kinase 2 (ErbB-2) amplification, microsatellite stability, and low tumor mutational burden of 6.3 mutations per megabase. And immunohistochemistry revealed that the tumor was programmed cell death-ligand 1 negative.

**Intervention:**

Zimberelimab monotherapy was accepted as third-line treatment.

**Outcomes:**

The patient had received zimberelimab for nearly 10 months, the best tumor response was PR (Response Evaluation Criteria in Solid Tumours) and no noticeable adverse reactions were observed.

**Lessons:**

PIK3CA-E542K, ErbB2 amplification, and SMAD4 mutations could be potential biomarkers for PD-1 inhibitors, but a single instance is insufficient to validate the hypotheses. A larger number of patients or more clinical data will be necessary to determine whether these gene mutations are appropriate biomarkers for patients when treatment with PD-1 inhibitors.

## 1. Introduction

Cervical cancer is a malignant tumor with a high incidence rate. It is estimated that 111,820 women will be diagnosed with cervical cancer in China by 2022, and 61,579 will die.^[1]^ Generally, chemoradiation and brachytherapy are recommended for patients with locally advanced diseases. Programmed cell death-ligand 1 (PD-L1)-positive recurrent or refractory cervical cancer patients are recommended a combination of bevacizumab and chemotherapy, with a median overall survival of up to 17 months.^[2,3]^

PD-1 (CD279) is mainly expressed on the surface of activated T cells. It is a member of the immunoglobulin superfamily and can bind to the PD-L1 to activate downstream signaling pathways, thereby inhibiting T cell activation.^[4]^ PD-1 blockade has shown efficacy in treating various cancers, including Hodgkin lymphoma and lung cancer.^[5]^ Existing predictive biomarkers for PD-1 blockade include PD-L1, tumor mutational burden (TMB), microsatellite instability-high (MSI-H), and mismatch repair deficiency (dMMR), which are recommended for various malignant tumors.^[6]^ However, in the phase 3 EMPOWER CERVICAL 1/GOG 3016/ENGOT cx9 trial, PD-L1–negative patients who underwent cemiplimab had a higher objective remission rate than those who underwent chemotherapy. This indicated that PD-L1–negative patients could benefit from immunotherapy, and there were other biomarkers that may predict immunotherapy efficacy, but they were not conclusive until now.

This case details the clinical experience of a biomarker-negative patient with recurrent metastatic cervical adenocarcinoma who, following curative surgery, received 2 lines of chemotherapy and radiation of metastases, has seen a long-term response to the PD-1 blockade Zimberelimab. We discuss other potential biomarkers that may predict immunotherapy efficacy in this case. Although there is no widespread consensus on other potential biomarkers besides PD-L1, TMB, MSI-H, and dMMR, we hope that it may provide another treatment option for patients with the same medical condition in the future.

## 2. Case presentation

The patient was born in 1970, diagnosed with cervical adenocarcinoma at the age of 41, and underwent hysterectomy after diagnosis, followed by adjuvant chemotherapy (4 cycles) and radiotherapy. Specific chemotherapy types and radiotherapy regimens have been lost.

During regular follow-ups, positron emission tomography and computed tomography (CT) in July 2020 demonstrated sporadic small nodules in both lungs, one in the middle lobe of the right lung and the other in the upper lobe of the left lung. The patient did not experience coughing, chest tightness, or any discomfort at that time. A subsequent percutaneous lung biopsy revealed an invasive adenocarcinoma. Metastatic adenocarcinoma of the cervical cancer was considered by integrating the medical history and immunohistochemistry results. The pathology report indicated transcription termination factor 1 (–), NapsinA (–), CK7 (+), CAM5.2 (+), P63 (–), P40 (–), CK5/6 (–), CaA (–), Syn (–), CD56 (–), Ki-67 (+60%). Pathological biopsy results suggested cervical adenocarcinoma with left lung metastases (pT0N0M1: FIGO Stage IVB).

As first-line treatment, the patient received bevacizumab (15 mg/kg of body weight), cisplatin (50 mg/per square meter body surface area), and paclitaxel (175 mg/square meter body surface area) for 6 cycles from August 3, 2020, to November 26, 2020. Unfortunately, carcinoembryonic antigen increased from 6.1 U/mL (March 2, 2021) to 13.66 U/mL (June 18, 2021) and ultimately to 27.28 U/mL (July 29, 2021). The CT scan on June 18, 2021, implied an increase in the size of lung metastases (the nodule in the right lung’s middle lobe measured 10 × 8 mm, while the nodule in the left lung’s upper lobe measured 25 × 20 mm) compared to pretreatment levels, which indicates progressive disease (PD).

Immunohistochemistry revealed that the tumor was PD-L1 negative. A 425-gene next-generation sequencing panel revealed PIK3CA E545K, SMAD4 1309-1G, and ALK E717K mutations, receptor tyrosine kinase 2 (ErbB2) amplification, a low TMB of 6.3 mutations per megabase, non–MSI-H, and mismatch repair-proficient (pMMR). Palliative radiotherapy (95% planning target volume = 50 Gy/10 F/2 w) for the nodule in the left lung was administered on June 28th (the other nodule in the right lung was tiny and did not meet palliative radiotherapy standards), followed by gemcitabine combined with cisplatin chemotherapy from August to September 2021 as second-line treatment. On the second day of the second chemotherapy cycle, the patient developed cold and experienced numbness and spasms in the extremities, which was considered to be due to cisplatin-induced neurotoxicity. During the third cycle of chemotherapy, the aspartate transaminase and alanine aminotransferase levels increased to 117 and 156 U/L, respectively. Furthermore, tumor response was identified as a partial response (PR) in September 2021 based on solid tumor evaluation standards (Response Evaluation Criteria in Solid Tumours [RECIST]1.1).

Zimberelimab was accepted as third-line treatment after communicating with her and her family. From September 30, 2021, to July 1, 2022, the patient was intravenously injected with 240 mg zimberelimab every 3 weeks (considering the patient’s tolerability) for 13 cycles. The latest CT was performed on August 19, and until now, the best tumor response was PR (RECIST1.1). Furthermore, the patient had a progression-free survival of more than 10 months with good tolerance. However, the patient became sick with mycotic pneumonia in late July, so anti-tumor therapy was discontinued, and the patient was treated symptomatically for a long time.

**Figure 1.**
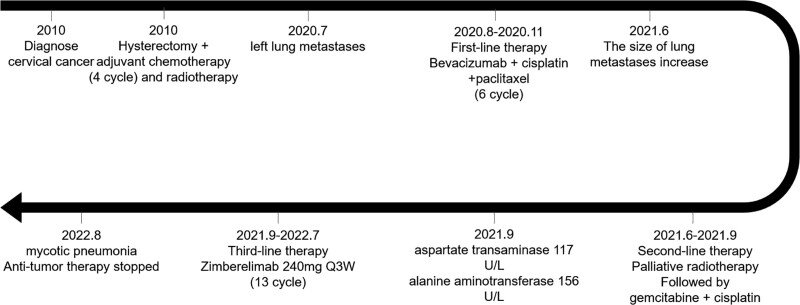
Whole treatment process of the patient.

**Figure 2.**
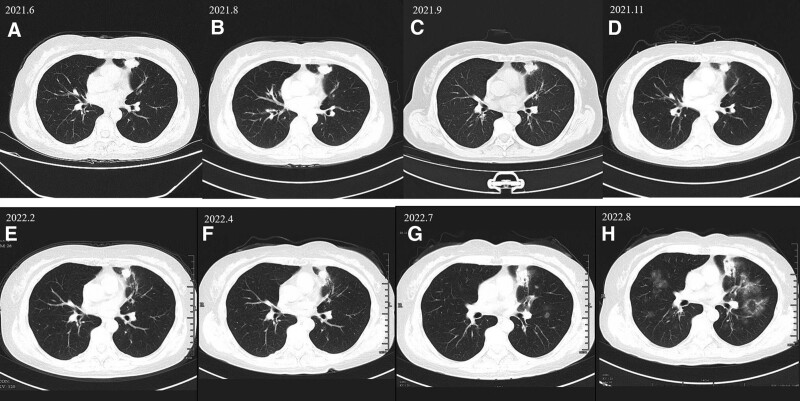
Results of chest CT scans before and after ICI treatment in a patient with cervical adenocarcinoma. (A) 2021.6.16. Tumor size: 25 × 20 mm. (B) 2021.8.3. The tumor measured 24 × 17 mm. (C) 2021.9.18. The tumor measured 23 × 17 mm. (D) 2021.11.26. The tumor measured 15 × 17 mm. (E) 2022.2.21. Tumor size:15 × 12 mm. (F) 2022.4.23. The tumor measured 15 × 13 mm. (G) 2022.7.16. The tumor measured 15 × 17 mm. (H) 2022.8.19. The tumor measured 15 × 17 mm. CT = computed tomography, ICI = immune checkpoint inhibitor.

**Figure 3.**
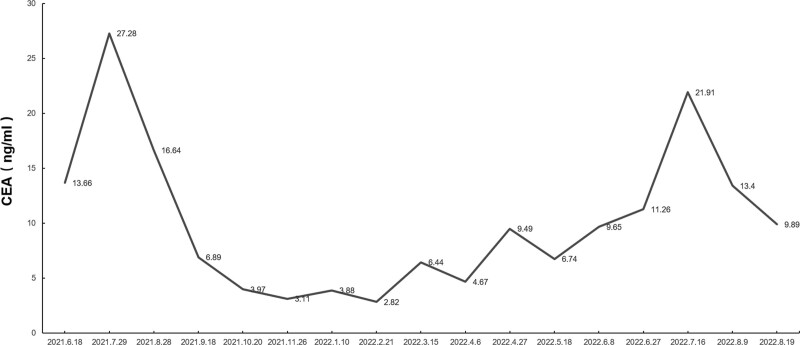
Serum CEA level for our patient before and after ICI treatment. CEA = carcinoembryonic antigen, ICI = immune checkpoint inhibitor.

Any potentially identifiable photographs or data included in this article were published with the individual’s written informed consent. The treatment process for the patient is shown in Figure 1. CT scans of tumor size during treatment are shown in Figure 2. Changes in carcinoembryonic antigen levels before and after immune checkpoint inhibitor treatment are shown in Figure 3.

## 3. Discussion

Cervical cancer has three most common histologic variants: adenocarcinoma, squamous cell carcinoma, and adenosquamous carcinoma. Approximately 20% of all cervical carcinomas are adenocarcinomas. Patients with adenocarcinoma exhibit a considerably worse chance of survival than those with squamous cell carcinoma, stage for stage.^[7]^

In our case, the patient was diagnosed with biomarker-negative cervical adenocarcinoma that progressed with previous treatment. Third-line treatment was challenging to determine at that time. Immunotherapy has improved the prognosis and survival of patients with cervical cancer. On October 13th, Pembrolizumab (Keytruda; Merck, USA) was approved by the Food and Drug Administration in 2021 for the treatment of patients with metastatic, persistent, or recurrent cervical cancer whose tumors express PD-L1 (CPS ≥ 1). Zimberelimab is a novel, completely human anti-PD-1 monoclonal antibody. In phase II clinical trials of cervical cancer, the objective response rate in patients with PD-L1–positive cervical cancer was 26.83%.^[8–10]^ However, the PD-L1–positive rate varies according to the histological subtype of cervical cancer. PD-1 positivity was found in 37.8% of squamous cell carcinomas, 28.6% of adenosquamous carcinomas, and 16.7% of endocervical adenocarcinomas in 1 investigation of cervical malignancies.^[3]^ Additionally, patients were enrolled in the phase 3 EMPOWER CERVICAL 1/GOG 3016/ ENGOT cx9 trial, regardless of their PD-L1 status. With cemiplimab, the median overall survival was 13.9 months (95% confidence interval [CI], 9.6–not evaluable) compared to 9.3 months (95% CI, 7.0–11.4) with chemotherapy in patients with PD-L1 positive (hazard ratio, 0.70; 95% CI, 0.46–1.05). In the subset of patients with PD-L1 negative, the median overall survival with cemiplimab was 7.7 months (95% CI, 4.3–12.3) versus 6.7 months (95% CI, 3.9–9.5) with chemotherapy (hazard ratio, 0.98; 95% CI, 0.59–1.62).^[11]^

All evidence suggests that immunotherapy is effective in patients with recurrent and metastatic cervical cancer with PD-L1 negativity. In our case, the patient was young and strongly desired to live. Immunotherapy was accepted as the eventual treatment. The patient had a long-term response after treatment with zimberelimab.

Why did immunotherapy benefit the present patient with negative biomarkers (PD-L1, MSI-H/dMMR, or TMB-H)? Prior radiation and chemotherapy may have a collaborative effect on immunotherapy. However, this patient may have been responsive to immunotherapy because of specific gene alterations.

Phosphatidylinositol-4,5-bisphosphate 3-kinase catalytic subunit alpha (PIK3CA) is a proto-oncogene that has attracted considerable attention in recent years. The P110 catalytic subunit of PI3K is encoded by the gene PIK3CA. Numerous cellular processes, such as cell growth and division (proliferation), migration, creation of new proteins, material transport inside cells, and cell survival, depend on PI3K signaling. Although genetic alterations in the PI3K/AKT pathway have been reported to be associated with immunotherapy resistance, emerging evidence has revealed that activation of this PI3K/AKT pathway might be a good target for immunotherapy. CLAP is a multicenter phase II study of patients with cervical cancer who have failed previous first-line or more therapies. The patient was treated with a combination of camrelizumab and apatinib. Subgroup analysis showed that patients with PIK3CA mutations had higher response rates than those without mutations. (61.9% vs 9.1%, OR = 16.25, 95% CI = 2.11–187.60, *P* = .004).^[12,13]^. According to the CLAP, PIK3CA mutations may predict better outcomes with immunotherapy. Another recent case suggests that the PI3KCA-E545K mutation enhances PD-L1 expression by upregulating PD-L1 transcription factor interferon regulatory factor 1 and that the PIK3CA mutation may be a biomarker for pembrolizumab treatment in cervical cancer.^[14]^

ErbB2 (ErbB2 Receptor Tyrosine Kinase 2, also known as HER2) encodes a protein. Its amplification contributes to the development and spread of various human malignancies. The ErbB2 amplification subgroup displayed increased ErbB2 protein expression, a higher abundance of tumor-infiltrating regulatory T cells, and a lower abundance of activated NK cells and CD8+ T cells compared to the standard ErbB2 control, according to the analysis of the Cancer Genome Atlas pan-cancer cohort data.^[15]^ Some preclinical and clinical investigations have revealed that Treg cells may impair individual immune surveillance against cancer, delay cancer patients from acquiring efficient antitumor immunity, and improve tumor growth.^[16]^ PD-1 blockade promotes the recovery of malfunctioning PD-1+/CD8+ T cells and improves PD-1 + Treg cell–mediated immunosuppression, which might explain how immunotherapy benefits ErbB2 amplification patients.^[17]^

Transforming growth factor β signaling also plays a vital role in cell growth, differentiation, apoptosis, migration, initiation, and progression of cancer.^[18]^ Studies have reported that SMAD4, a central mediator of transforming growth factor-β signaling, is mutated or deleted in 20% of pancreatic ductal adenocarcinomas, significantly affecting cancer development. SMAD4 deficiency increases tumor cell immunogenicity by enhancing spontaneous DNA damage and boosting sting-mediated type I interferon signaling. This may stimulate type 1 conventional dendritic cells (cDC1) and CD8 + T cells, allowing for tumor control.^[19]^ SMAD4 mutations may cause smad4 deficiency, and SMAD4 deficiency might increase the immune prototype, leading to an extended response to PD-1 blockade.

Currently, there is no research or report on the connection between the ALK-E171K mutation and the benefit of immunotherapy. Patients with anaplastic lymphoma kinase (ALK) mutations (mostly ALK rearrangements) were excluded from clinical immunotherapy studies. In addition, no individuals with ALK mutations have benefited from immunotherapy in the few studies that have been undertaken.^[20,21]^ Therefore, ALK-E171K mutations are less likely to serve as biomarkers for immunotherapy.

This case details the clinical experience of a biomarker-negative patient with recurrent metastatic cervical adenocarcinoma who, following curative surgery, received 2 lines of chemotherapy and radiation of metastases, has seen a long-term response to the PD-1 blockade Zimberelimab, the best tumor response was PR (RECIST1.1) and no noticeable adverse reactions were observed. We discuss other potential biomarkers that may predict immunotherapy efficacy. Through summary and analysis of previous literature, we think potential biomarkers could be PIK3CA-E542K, ErbB2 amplification, or SMAD4 mutations.

PI3KCA-E545K mutation enhances PD-L1 expression by upregulating PD-L1 transcription factor interferon regulatory factor 1 and that the PIK3CA mutation could be a biomarker for PD-1 blockade treatment in cervical cancer. ErbB2 amplification showed a high abundance of tumor-infiltrating regulatory T cells and a low abundance of activated NK cells and CD8 + T cells. PD-1 blockade promotes the recovery of malfunctioning PD-1+/CD8+ T cells and improves PD-1 + Treg cell–mediated immunosuppression, which might explain how immunotherapy benefits ErbB2 amplification patients. SMAD4 mutations may cause SMAD4 deficiency, and SMAD4 deficiency might increase the immune prototype, leading to an extended response to PD-1 blockade.

But there were limitations in the case, this study is based on a single case, and a single instance is insufficient to validate the hypotheses. A larger number of patients or more clinical data will be necessary to determine whether these gene mutations are appropriate biomarkers for patients when treatment with PD-1 inhibitors. Hopefully, from the description of this case, patients with PD-L1 negative, pMMR, non-MSI-H, and TMB-low cervical adenocarcinoma who are incapable of chemotherapy and have particular gene mutations may benefit from immunotherapy.

## Author contributions

**Methodology:** Jun Chen.

**Validation:** Jun Chen.

**Formal analysis:** Linrong Pang.

**Writing—review & editing:** Lianxiang He, Ting Li, Xiaochun Cheng.

**Conceptualization:** Ting Li, Xiaochun Cheng.

**Writing—original draft:** Ting Li.
